# Bioequivalence of long-chain omega-3 polyunsaturated fatty acids from foods enriched with a novel vegetable-based omega-3 delivery system compared to gel capsules: a randomized controlled cross-over acute trial

**DOI:** 10.1007/s00394-021-02795-7

**Published:** 2022-01-18

**Authors:** Welma Stonehouse, Bradley Klingner, Rachel Tso, Pey Sze Teo, Netsanet Shiferaw Terefe, Ciarán G. Forde

**Affiliations:** 1 Commonwealth Scientific Industrial Research Organisation (CSIRO), Health and Biosecurity, PO Box 10041, Adelaide, BC, SA 5000 Australia; 2 Clinical Nutrition Research Centre, A*STAR Singapore Institute of Food and Biotechnology Innovation, Singapore, Singapore; 3 Commonwealth Scientific Industrial Research Organisation (CSIRO), Agriculture and Food, Werribee, VIC Australia; 4 Department of Physiology, Yong Loo Lin School of Medicine, National University of Singapore, Singapore, Singapore; 5 Present Address: Sensory Science and Eating Behaviour, Division of Human Nutrition and Health, Wageningen University, Wageningen, The Netherlands

**Keywords:** Omega-3 long-chain polyunsaturated fatty acids, Docosahexaenoic acid, Bioavailability, Bioequivalence, Vegetable-based delivery system, Algal oil

## Abstract

**Purpose:**

To evaluate bioavailability of omega-3 long-chain polyunsaturated fatty acids (LCPUFA) from foods enriched with novel vegetable-based encapsulated algal oil across Australian and Singaporean populations.

**Methods:**

27 men (*n* = 12 Australian European; *n* = 15 Singaporean Chinese), 21–50 yr; 18–27.5 kg/m^2^, with low habitual intake of omega-3 LCPUFA completed a multicentre randomised controlled acute 3-way cross-over single-blind trial. They consumed, in random order 1-week apart after an overnight fast, standard breakfast meals including 400 mg docosahexanoic acid (DHA) from either extruded rice snacks or soup both containing cauliflower-encapsulated HiDHA^®^ algal oil or gel capsules containing HiDHA^®^ algal oil. Blood samples for analysis of plasma DHA and eicosapentaenoic acid (EPA) were taken pre-meal and after 2, 4, 6, 8 and 24 h. Primary analyses comparing 24-h incremental area under the plasma DHA, EPA and DHA + EPA concentration (µg/ml) curves (iAUC_0-24 h_) between test foods were performed using linear mixed models by including ethnicity as an interaction term.

**Results:**

Plasma iAUC_0-24 h_ did not differ significantly between test foods (adjusted mean [95% CI] plasma DHA + EPA: extruded rice snack, 8391 [5550, 11233] µg/mL*hour; soup, 8862 [6021, 11704] µg/mL*hour; capsules, 11,068 [8226, 13910] µg/mL*hour, *P* = 0.31) and did not differ significantly between Australian European and Singaporean Chinese (treatment*ethnicity interaction, *P* = 0.43).

**Conclusion:**

The vegetable-based omega-3 LCPUFA delivery system did not affect bioavailability of omega-3 LCPUFA in healthy young Australian and Singaporean men as assessed after a single meal over 24 h, nor was bioavailability affected by ethnicity. This novel delivery system may be an effective way to fortify foods/beverages with omega-3 LCPUFA.

**Trial registration:**

The trial was registered with clinicaltrials.gov (NCT04610983), date of registration, 22 November 2020.

**Supplementary Information:**

The online version contains supplementary material available at 10.1007/s00394-021-02795-7.

## Introduction

Non-communicable diseases (NCD) in both Australia and Singapore account for significant proportions of deaths, disease burden and hospitalisations, with poor diet quality as the major contributor to the NCD burden [1–3]. Marine-derived omega-3 long-chain polyunsaturated fatty acids (LCPUFA) (particularly eicosapentaenoic acid (EPA) and docosahexaenoic acid (DHA)) are associated with numerous health benefits throughout the life cycle. For example, Omega-3 Index levels (O3I, a measure of the proportion of DHA and EPA of total fatty acids in erythrocytes) of 8% *vs*. 4% were related to a 30% reduced risk of fatal coronary heart disease (CHD) [4]. More recently, O3I levels of 8% have also been linked to increased longevity, reduced all-cause mortality, improved depressive symptoms, reduced arthritis risk and improved cognitive function [5–11]. Despite the well-established health benefits of omega-3 LCPUFA, intakes in Western and most Asian diets are below recommendations [12, 13]. Low levels of consumption have been associated with an increased risk of cardiovascular mortality in a Chinese population [12]. Consumption of ~ 2–3 serves/week of fish, including oily fish, is recommended to achieve ~ 250 – 500 mg/day of combined EPA + DHA, and up to 1 g/day for those with CHD [14–17]. Achieving these intakes through dietary means is challenging, particularly for individuals who do not consume fish or seafood — the major dietary sources of omega-3 LCPUFA [8]. Vegans and vegetarians are particularly at risk. Several studies have shown that vegan diets are devoid of DHA and vegetarian diets that include dairy products and eggs only provide about 0.02 g DHA/day [18]. These low intakes were accompanied by substantially lower levels of DHA in plasma, serum, red blood cells (RBC) and plasma phospholipids (PL) in vegans and vegetarians compared to omnivores [18]. Thus, strategies such as fortifying commonly consumed foods and beverages that can be easily incorporated into the diet with omega-3 LCPUFA, will assist consumers in achieving their omega-3 LCPUFA intake targets and potentially contribute to positive health impacts.

Some of the challenges in the production of foods fortified with omega-3 LCPUFA include their undesirable odour and taste and their susceptibility to oxidative degradation [19]. A recently developed novel vegetable-based encapsulation system for delivery of omega-3 LCPUFA has been shown to provide superior protection against oxidation of omega-3 LCPUFA compared to other commercially available microencapsulation techniques [20]. However, the vegetable carrier matrix that is high in fibre and various bio-actives, differs considerably from that of the usual omega-3 LCPUFA matrix, namely seafood and oil-based supplements, and it is unknown whether this matrix may impact the release and bioavailability of omega-3 LCPUFA in vivo*.* Bioavailability is the extent to which a nutrient can be absorbed and transported to the systemic circulation or the site of physiological activity and hence any health effects depend on the bioavailability of the active agent [21]. Evidence for the effects of food fortification delivery systems on omega-3 LCPUFA bioavailability is limited and there is no evidence for the effect of a vegetable-based delivery system. Some evidence suggests that omega-3 LCPUFA bioavailability is not affected by microencapsulation delivery techniques [19, 22–24] or food structure, e.g. fish flesh vs. fish oil [25–27], while bioavailability may be enhanced by factors, such as co-consumption of omega-3 LCPUFA with antioxidant-rich supplements [28, 29], a background diet lower in omega-6 PUFA [30], emulsification [31], increased dose and chemical form (e.g. triacylglycerol *vs*. ethyl esters or phospholipids *vs*. triacylglycerol) [32–34] of omega-3 LCPUFA. Furthermore, it is important to test the bioavailability of active agents from novel products within the populations that the products are targeted for. It is a possibility that inter-ethnic differences exist in omega-3 bioavailability [35], but this hypothesis remains untested.

The aim of the study was to evaluate the bioavailability of omega-3 LCPUFA from foods enriched with vegetable-encapsulated algal oil across Australian and Singaporean populations. It was hypothesised that delivery of omega-3 LCPUFA with vegetables as carriers will not impact its bioavailability; and bioavailability of omega-3 LCPUFA will not be influenced by inter-ethnic differences. The primary outcomes were the 24-h incremental area under the plasma DHA, EPA and DHA + EPA concentration (µg/mL) curves (iAUC_0-24 h_), which served as a measure of bioavailability [21]. Secondary outcomes were time to maximal value (Tmax) and maximal value (Cmax) on the plasma DHA, EPA and DHA + EPA concentration (µg/mL) curves.

## Methods

The clinical trial was conducted at CSIRO Nutrition and Health Research Clinic, Adelaide, South Australia and A*STAR Clinical Nutrition Research Centre in Singapore.

Human Research Ethics Committee approvals were obtained from the CSIRO Human Research Ethics Committee, Adelaide, South Australia (reference number 2020_026_HREC) and the National Healthcare Group Domain Specific Review Board, Singapore (reference number 2020/00269). Oral and written information about the study objectives and protocol were provided to each individual and written informed consent obtained prior to performing any study-related assessments. The trial was prospectively registered with clinicaltrials.gov (NCT04610983). The trial was conducted in accordance with International Council on Harmonisation Good Clinical Practice (ICH-GCP) guidelines. The intervention phase of the study was executed from 1 to 25 February 2021 in Adelaide and 1 December 2020 to 10 February 2021 in Singapore.

### Participants

A total of 27 (*n* = 12 in Australia; *n* = 15 in Singapore) apparently healthy men aged 21–50 years inclusive with a body mass index between 18 and 27.5 kg/m^2^ were recruited. Men were chosen as the study population to ensure a metabolically homogenous sample and gender has been shown to predict circulatory omega-3 LCPUFA levels [32]. Participants were not allowed to consume fish oil supplements or ≥ 2 fatty fish meals per week over the past 3 months; and they had to identify as either Australian European (for the Australian cohort) or Singaporean Chinese (for the Singaporean cohort).

Exclusion criteria included: history of chronic disease, e.g. cancer, type 2 diabetes, cardiovascular disease (CVD), liver disease or any condition that may, in the opinion of the principal investigator, have influenced the study outcomes; history of gastrointestinal disease, pancreatic insufficiency, conditions resulting in fat malabsorption (e.g. chronic pancreatitis, cystic fibrosis, coeliac disease, Crohn's disease, gastric bypass surgery, small bowel resection, abnormal thyroid function); bleeding disorders, taking anticoagulants or received anticoagulants within 28 days of day 1 of the trial, with the exception of low dose aspirin up to 150 mg daily; any medical procedures deemed by the principal investigator to affect study outcomes; known food allergies, hypersensitivity, dietary avoidance or intolerance to the study foods; taking medications/supplements known to influence lipid metabolism and gastric emptying; on any weight loss program; history of smoking during the 6 months prior to the study; persons considered by the investigator to be unwilling, unlikely or unable to comprehend or comply with the study protocol; history of drug abuse or alcoholism; participation in another research study within 30 days preceding the start of the trial.

### Study design and procedures

A multicentre, randomised, controlled, acute, 3-way cross-over single-blind study design was used. Participants attended a screening visit to confirm their eligibility for inclusion into the trial. Participants were randomly assigned by computer sequence generation (http://www.randomisation.com) to 1 of 6 treatment sequences in Australia or 1 of 4 treatment sequences in Singapore. The random allocation sequences were generated by staff members not involved with entering participants into the trial to ensure allocation concealment. Laboratory staff responsible for analysing blood samples and staff performing statistical analyses were blind to treatment allocations until after a statistical analyses report was delivered. However, because of the appearance of the study products, participants and staff administering the products could not be blinded.

Following screening and enrolment, participants attended the research clinic on 3 occasions at least 1 week apart during which they consumed 1 of 3 test products according to the treatment sequence they were assigned to; either a snack or soup, both containing cauliflower-encapsulated HiDHA® algal oil or gel capsules containing HiDHA® algal oil (control). A 1-week washout period has been shown to be sufficient for plasma samples to return to baseline [36].

Participants arrived at the research clinic after an overnight fast (> 10 h). Weight, percentage body fat mass (measured at screening in Singaporean participants) and blood pressure were measured, an intravenous cannula was inserted into the participants’ arm for a duration of 8 h during which the participant remained at the research clinic. A fasting blood sample (4 mL) was collected after which participants consumed the test product with a standardised breakfast within 15 min. Subsequent blood samples were collected after 2, 4, 6 and 8 h. The cannula was removed, and participants left the research clinic to return the next morning after an overnight fast for collection of the 24-h sample. The period of 24 h was chosen as it represents an intake interval of daily intake of omega-3 supplements [36]. Throughout the ~ 9-h day at the clinic, participants had access to low-fat snacks and a lunch meal devoid of omega-3 LCPUFA that they could consume ad libitum. Participants left the clinic with a take-home low-fat dinner meal and low-fat snacks, all of which they could consume ad libitum.

Apart from the study-specific test products, participants were requested to maintain their habitual lifestyle patterns throughout the duration of the study and to avoid fatty fish and omega-3 LCPUFA-containing supplements. Participants were provided with a checklist to record any non-compliance or accidental consumption of these foods and returned the checklist at their subsequent visit.

### Test products and dietary intakes during test occasions

Test meals included:

- Soup (semi-solid food) – 4.2 g cauliflower-encapsulated HiDHA^®^ algal oil with 25% oil loading (providing 400 mg DHA and 13.6 mg EPA) powder was mixed into a 200 g serving of soup (commercially available Continental® cream of chicken packet-soup) + standardised breakfast.
- Extruded rice snack (solid food) – a 58.2 g serving barbecue tangy flavored extruded rice snack using cauliflower-encapsulated HiDHA^®^ algal oil as an ingredient (providing 400 mg DHA + 13.8 mg EPA) + standardised breakfast.
- Gel capsule (control) – 2 × 0.5 g algal oil gel capsules containing HiDHA^®^ algal oil providing 400 mg DHA + 14.7 mg EPA) + standardised breakfast. Bovine-derived empty gelatine capsules (Surgipack^®^) were purchased from a local pharmacy and filled with 0.5 g algal oil each.

The serving size of the test products were based on the amount needed to provide 400 mg DHA.

The standardised breakfast consisted of a small carton of Milo (Nestle: 200 ml), an apple (200 g) and one muesli bar (Uncle Toby’s, 31 g). The total energy content was ~ 1600 kJ (~ 382 kcal) and total fat content was 7.3 g. Throughout the 8-h day at the clinic, participants had access to low-fat snacks and a lunch meal devoid of omega-3 LCPUFA that they could consume ad libitum*.* Low-fat was defined as ≤ 3 g fat/100 g solid food or ≤ 1.5 g fat/100 ml for liquid food [37, 38]. Snacks included rice crackers, fruit, low-fat dairy products (yoghurt, chocolate milk), fruit juice, tea, coffee and water. Lunch and dinner meals were low-fat frozen meals with similar nutrient profiles, selected from the Lean Cuisine Steam range in Australia (https://www.vescofoods.com.au/brands/lean-cuisine) and the CP Gourmet Ready Meals range in Singapore (https://www.cpshopz.sg/brands/cp/ready-meal). Breakfast, which was either a selection of cereals or a carton of Milo, apple, and muesli bar, was provided to participants after collection of their 24-h blood sample.

### Production of vegetable-encapsulated HiDHA® algal oil powder and extruded savoury snack

The HiDHA® algal oil was purchased from NuMega Ingredients Pty Ltd, Melbourne, Victoria, Australia. Cauliflower was obtained from a local farm (Freshselect, Werribee, Australia). All the other ingredients were purchased from accredited food ingredient suppliers and retailers. The production of the cauliflower-encapsulated HiDHA^®^ algal oil powders was conducted in accordance with the process described in [20]. The process involved washing and sanitization of cauliflower heads, heat treatment at 100 ˚C for 15 min to inactivate spoilage and pathogenic microorganisms and quality-degrading enzymes, size reduction and pureeing of the cauliflower heads using Comitrol® processor (Urschel, USA) and a Tri-blender^®^ (TECNinox Srl, Italy), addition of HiDHA algal oil to the puree at 1:3 oil to vegetable solids ratio, pre-emulsification of the oil-puree mixture using a Tri-blender^®^ (TECNinox Srl, Italy), emulsification using a two stage high pressure homogenizer (APV Rannie 30.60, SPXFLOW, NC, USA), and freeze-drying of the emulsion into powder. The HiDHA^®^ algal oil was added to the cauliflower puree under N_2_ blanketing to protect the oil from oxidation. Following drying, the encapsulated oil powder was packed in triple laminated aluminum foil pouches with nitrogen flushing and was stored at 4 ˚C or lower until use. For the clinical trial, 4.2 g aliquots of the powder were packed in single use triple laminated aluminum foil pouches with nitrogen flushing and were stored at 4 ˚C or lower until use.

The extruded snack blend was composed of rice flour base, cauliflower powder and cauliflower algal oil powder formulated to provide 400 mg of DHA per 50 g of dry non-flavored extrudate. Extrusion was conducted using a lab-scale co-rotating twin-screw extruder (DSE32-II, Jinan Kredit Machinery Co., Ltd, Shangdong, China). The temperature profile along the barrel from feed to die were set to 30, 80, 120 and 150 ℃ respectively. The feed rate and screw speed were fixed at 15 kg/hr and 300 rpm, respectively. The feed moisture content was adjusted to around 22% (wet basis). Following extrusion, the extrudate was oven-dried at 85 ˚C for one hour to an average moisture content of 2.2%. After overnight storage at 4 ˚C, the extrudate was flavored with barbecue tangy seasoning (The Product Makers, Melbourne, Australia). The flavoring involved preheating the extrudates in an oven for 3 min at 130 ˚C, spraying canola oil on the pre-heated extrudate while tumbling continuously to ensure that the surface of the extrudates is uniformly coated with oil, sprinkling the seasoning, tumbling and a final heating step in an oven for 2 min at 120 ˚C, followed by cooling and packing in triple laminated aluminum foil pouches. The flavoring raised the weight of the snacks by ~ 16.4%, increasing the serving size from 50 g to 58.2 g. The extruded flavored samples were stored at 4 ˚C or lower until consumption.

The cauliflower oil powder and the test products were manufactured in facilities certified for production of food for human consumption in accordance with an approved Hazard Analysis Critical Control Point (HACCP) plan. Only food grade ingredients were used in the production process. All the equipment used in the production process were sanitised, swabbed for microbial contamination and re-cleaned until they became suitable for food contact. All the processing steps, from the purchase of the ingredients to the processing and handling conditions at each processing stage were recorded in a process log sheet. The microbial and physicochemical qualities of the final products were tested in accordance with the approved HACCP plan. The products were released as fit for human consumption after CSIRO’s Food Risk Assessment Team examined the process log sheet and the results of the microbial and physicochemical analyses.

### Clinical assessments

Medical and surgical history, medication, supplement use and demographic information were obtained by questionnaire and interview of participants.

Height and weight were measured, and BMI calculated using the formula: weight (kg)/height (m)^2^. Bioelectrical impedance analysis (BIA) was used to assess % body fat mass using a multi-frequency BIA with 8 tactile electrodes (InBody 230, Biospace Co. Ltd, Seoul in Australia and Tanita BC-418, Tokyo in Singapore). Measurements were obtained after voiding. Participants stood upright, positioned their bare feet on the footpads and their hands on the handles. A small electrical current was passed through the body, resistance was measured, and total body water and the corresponding body composition measures were calculated by the inbuilt software.

Resting blood pressure was measured with participants in a seated position using an automated blood pressure monitor (InBody BPBIO750 Blood Pressure Monitor in Australia and Omron Blood Pressure Monitor, HEM-907 in Singapore). Body temperature was measured using a digital tympanic thermometer or digital infrared forehead thermometer.

### Fatty acid analysis of test products

The analysis of DHA and EPA in HiDHA algal oil and test products involved fatty acid methyl esterification (FAME) followed by gas chromatography (GC) analysis to quantify the fatty acids in the products. The extruded snack samples were powdered using mortar and pestle prior to analysis. A previously reported method [39] was used after modification for direct methylation of the cauliflower-encapsulated oil and the extruded snack powders. Accordingly, 75 μL internal standard solution (17:0 Triheptadecanoin, TAG) 10 mg/ml in toluene) was added to (10 ± 0.01) mg of the powder (cauliflower oil and extruded snack powder) samples. The mixtures were resuspended in 0.9 mL 1 N methanolic HCl and 0.1 mL of Dichloromethane in argon-flushed 2 mL GC vials and were incubated in a shaking water bath at 80 °C for 2 h. Following incubation, the vials were remixed and were cooled down to room temperature. Then 0.3 mL NaCl was added to the vials and the FAME were extracted using 0.3 mL hexane. Prior to injection to GC for quantification, 0.3 mL of hexane was added to the FAME samples in accordance with the method described in [40]. The FAME solution (1 µL) was injected into a GC column (BPX 70 fused silica column, 30 m, 0.25 mm id and 0.25 μm films, SGE, Australia) at a split ratio of 1:50. An Agilent GC system (model 7890A, Agilent Technologies Australia Pty Ltd., Victoria, Australia) equipped with a model 7693 auto-sampler was used for the analysis. The GC column temperature was increased from 150 to 210 °C at a rate of 3 °C/min, and further increased at a rate of 50 °C/min to a final temperature of 240 °C. The injector and detector (FID) were maintained at 240 and 250 °C, respectively. Agilent Chemstation software [B.04.02 SP2 (256)] was used for integrating the peak areas. The peaks were identified based on the retention times of the reference fatty acid methyl ester standards. The quantity of EPA and DHA in the samples (mg fatty acid/g dry weight) were calculated based on the AOCS official method [41]. The oil samples were methyl esterified and analysed in the same way except that 50 µL of the internal standard solution was mixed with 5 ± 0.1 mg of the oil samples. The methylation and analyses of all the samples were conducted in triplicates.

### Plasma fatty acid analysis

Venous blood samples were collected into vacutainers containing EDTA (purple top). Plasma was prepared by centrifugation at 2800 g for 15 min at 4 °C. The resulting plasma was aliquoted and stored in  − 80 °C freezers until analysis. The Australian samples were analysed by the CSIRO Analytical Laboratory and the Singaporean samples by the Duke-NUS Metabolomics Laboratory, Singapore using the same methodology.

Samples from each participant were analysed within the same analytic run to reduce variation. Plasma fatty acid concentrations (µg/mL) were analysed using a GC technique. A 100 µL plasma sample was treated with 1 mL 0.2 M KOH/MeOH (0.005% BHT) and an internal standard (Australia: 40 µL of a 0.63 mM solution of C23:0 (internal standard; 450uL of 7.05E-3 tricosanoic acid [C23:0 acid, NU-CHEK-PREP, INC No.N-23-A] in 5 ml chloroform; Singapore: 50µL of internal standard (26.44 mg triheptadecanoin [C17:0 triglyceride, Sigma No. T-2151 99%]) in 25 ml chloroform (equivalent to 1.0 mg/ml of C17:0 fatty acid)). This was followed by 10 min of heating at 90 °C; addition of 2 mL methanol; acidification with 200 µL of acetyl chloride; heating at 90 °C for 1 h while vortexing approximately every 10 min after which the samples were returned to room temperature. 1.5 ml hexane was added and the sample shaken using the IKA vibrax-VXR orbital shaker for 30 s after which the sample was centrifuged at 2095 g for 10 min. The hexane top layer was extracted being careful not to disturb the bottom layer and reconstituted to 100 µL. A 1.0 µl aliquot was then injected onto a gas chromatographic column (DB – FastFAME, 20 m × 0.18 mm from Agilent Technologies), using Agilent Technologies 7890A with a split 50:1 injector. Fatty acids were identified by comparison with authentic Sepelco 37 component FAME mix (Sigma-Aldrich, Australia) and verified using EPA and DHA standards. Peaks were measured using OpenLAB CDS Chemstation software and fatty acids were calculated against the internal standard. The intra-assay and inter-assay coefficient of variations were: for Australia, 5.8% and 12.1% for DHA, respectively and 7.7% and 12.2% for EPA, respectively; for Singapore, 12.5% and 12.9% for DHA, respectively and 15.1% and 11.3% for EPA, respectively.

### Statistical analysis

It was calculated that a sample size of *n* = 10 per site would be required to provide > 80% power to detect a difference of 10% in iAUC [31]. To account for a 20% attrition rate, at least 12 participants per site had to be recruited.

The 24-h incremental area under the curve (iAUC_0-24 h_) was calculated for plasma DHA, EPA and EPA + DHA (primary outcomes) using the trapezoidal rule [42]. Concentrations that fell below T_0_ concentrations were excluded from the calculation. Time to maximal value (Tmax) and maximal value (Cmax) were determined for plasma DHA, EPA and EPA + DHA.

Blinding to treatment allocations was maintained throughout statistical analyses. Outcome variables were examined for normality using a combination of Kolmogorov–Smirnov, Shapiro–Wilk tests and observing normality plots. All randomised participants’ data were included in the analyses as no valid reason could be provided for excluding outliers. For continuous variables, descriptive summaries included means and SD for normally distributed variables or medians and 25 and 75 percentiles for non-normally distributed variables. Estimates of treatment effects are presented as adjusted means and 95% CIs.

Primary analyses, comparing iAUC_0-24 h_ for plasma DHA, EPA and DHA + EPA between test foods were performed using linear mixed models including treatment as fixed effect and controlling for ethnicity (Australian European and Singaporean Chinese) and Time_0_ for the respective variable and treatment. Interaction terms were included for treatment*ethnicity to assess evidence of effect modification by ethnicity; and for treatment*allocation order to assess whether the order in which participants received the study products affected responses. A compound symmetry repeated covariance matrix structure was used and a random intercept per participant included. As no significant treatment*ethnicity or treatment*allocation order interactions were observed, these interaction terms were removed from the model. The same statistical analyses methods were used to analyse differences between study products for plasma DHA, EPA and DHA + EPA Cmax. As Tmax data were non-parametric, comparisons between treatments were performed using the Friedman Test for related samples.

For analyses to compare postprandial plasma DHA, EPA and DHA + EPA concentrations between study products over 24 h, changes from Time_0_ were calculated by subtracting Time_0_ data from 2-, 4-, 6-, 8- and 24-h data. Analyses to compare study products were performed using linear mixed models including treatment and time as fixed factors and treatment*time as interaction term while controlling for ethnicity and Time_0_ for the respective variable and treatment. To assess whether responses between treatments were different between ethnic groups, an interaction term was included for treatment*time*ethnicity. An interaction term for treatment*time*allocation order was also included. As no significant treatment*time*allocation order interactions were observed, this interaction term was removed from the model. But as a significant interaction was shown for treatment*time*ethnicity, statistical analyses were performed stratified by ethnic group. A first-order autoregressive (AR(1)) repeated covariance type was used and a random intercept per participant included. Post-hoc analyses were performed if significant treatment*time interactions were observed, using repeated measures general linear model with Bonferroni adjustments.

Assumptions underpinning mixed linear statistical analyses, namely normal distribution and homoscedasticity of residuals, were met for all tests. The threshold for significance was set at 0.05 (two-sided). Statistical analyses were performed using SPSS software, version 26 (IBM Corporation, New York, USA).

## Results

### Study population

**Fig. 1:**
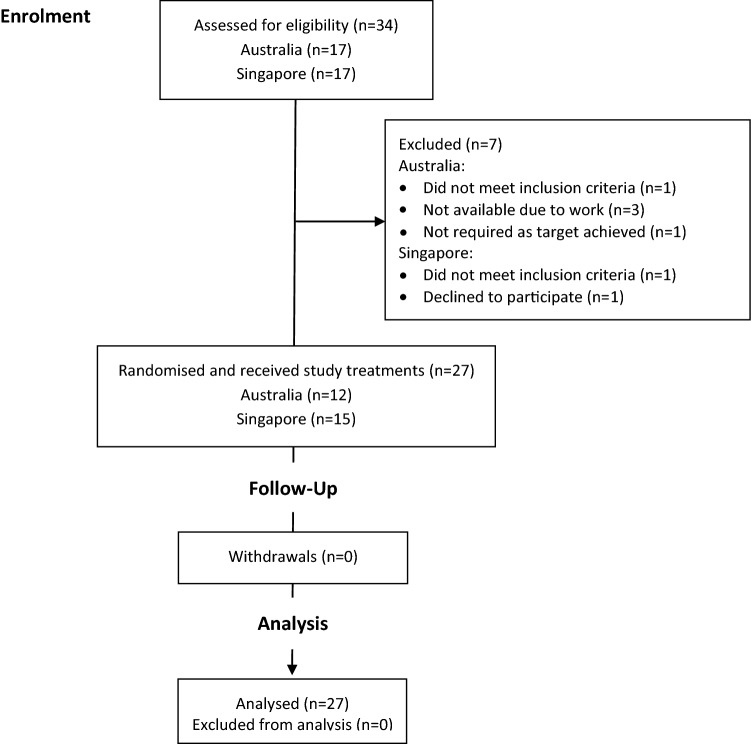
Flow chart of participants through the trial

A total of 34 participants were assessed for eligibility (Fig. 1) and 27 enrolled (Australia: *n* = 12; Singapore: *n* = 15). No participants withdrew or were lost to follow-up. Baseline characteristics summarised in Table 1 reflect inclusion criteria, namely healthy, normal weight men, aged 21–50 years.

**Table 1:** Baseline characteristics

	Total group (*n* = 27)		Australian European (*n* = 12)		Singaporean Chinese (*n* = 15)	
	Mean	SD	Mean	SD	Mean	SD
Age (years)	33.1	9.18	38.6	8.98	28.7	6.77
Systolic BP (mmHg)	119	8	116	9	121	6
Diastolic BP (mmHg)	74	8	73	9	75	8
Height (cm)	176	7.9	180	7.4	172	6.5
Weight (kg)	71.1	11.2	77.3	11.6	66.1	8.2
BMI (kg/m^2^)	22.9	2.60	23.8	2.47	22.1	2.54
BIA Body Fat %	16.3	5.65	17.3	5.69	15.6	5.70

*BIA* bioelectrical impedance analysis; *BMI* body mass index; *BP* blood pressure

### Plasma fatty acids

**Fig. 2:**
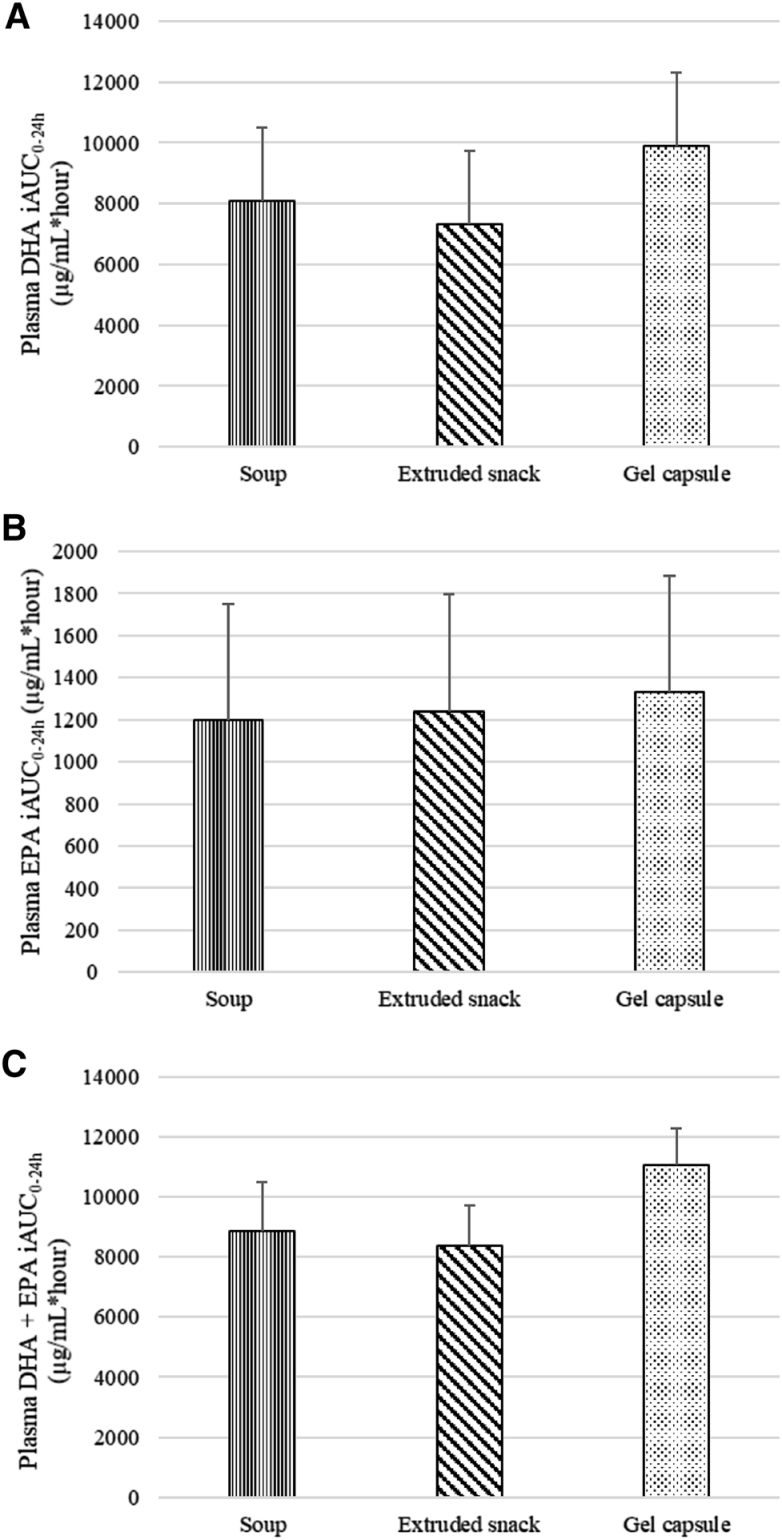
Adjusted mean (95% CI) incremental area under the curve (iAUC_0-24 h_) for plasma DHA (**A**), EPA (**B**) and DHA + EPA (**C**) by treatment. DHA, docosahexaenoic acid; EPA, eicosapentaenoic acid; iAUC_0-24 h_; incremental area under the curve over 24 h. Comparisons between treatments were performed using linear mixed effects models (*n* = 27) including treatment as fixed term while controlling for ethnicity and Time_0_ for the respective variable and treatment. Treatments did not differ significantly (*P* = 0.31). Treatment*ethnicity and treatment*allocation order interactions were not significant (*P* > 0.05)

Descriptive statistics estimated from raw data for DHA, EPA and DHA + EPA iAUC_0-24 h_, Cmax and Tmax are summarised in **Supplemental **Table 1. DHA, EPA, DHA + EPA iAUC_0-24 h_ (Fig. 2), Cmax and Tmax (Table 2) did not significantly differ between study products and the effects were also not significantly different between Australian European and Singaporean Chinese groups (treatment*ethnicity interaction, P = 0.43). **Supplemental **Fig. 1 provides box plots to illustrate the overall patterns in plasma DHA + EPA iAUC_0-24 h_ responses to study products. The box plots show large variation between participants and overlap in responses between study products. The outliers (A4 and A7) were likely responsible for skewing the average values presented in Fig. 2, making it appear as if the iAUC for gel capsules was larger when it was not. **Supplemental **Fig. 2 illustrates individual participants’ responses in plasma DHA + EPA iAUC_0-24 h_, further demonstrating no apparent trends for any specific study product. The iAUC_0-24 h_ for Singaporean Chinese appeared to be lower than that of Australian European. However, as different laboratories were used for the plasma EPA and DHA analysis, no conclusions can be made from this observation.

**Table 2:** Cmax and Tmax for plasma DHA, EPA and EPA + DHA by treatment

	Soup		Extruded rice snack		Gel capsule		*P* value^a^
	Mean	95% CI	Mean	95% CI	Mean	95% CI	
DHA Cmax (µg/mL)	52.4	[50.0, 54.9]	51.5	[49.1, 54.0]	55.2	[52.8, 57.6]	0.06
EPA Cmax (µg/mL)	18.5	[17.9, 19.1]	18.4	[17.8, 19.0]	18.6	[18.0, 19.2]	0.86
DHA + EPA Cmax (µg/mL)	70.5	[67.7, 73.3]	69.6	[66.8, 72.4]	73.5	[70.6, 76.3]	0.12

	Median	25, 75 Percentile	Median	25, 75 Percentile	Median	25, 75 Percentile	*P* value^b^
DHA Tmax (hours)	4	[2, 8]	4	[2, 6]	6	[4, 6]	0.12
EPA Tmax (hours)	6	[2, 8]	6	[2, 8]	6	[4, 8]	0.50
DHA + EPA Tmax (hours)	4	[4, 8]	4	[2, 6]	6	[4, 6]	0.50

DHA, docosahexaenoic acid; EPA, eicosapentaenoic acid; Cmax, maximal concentration; Tmax, time to maximal concentration

^a^Comparisons between treatments were performed using linear mixed effects models (*n* = 27) including treatment as fixed effect while controlling for ethnic and Time_0_ for the respective variable and treatment. Treatment*ethnicity and treatment*allocation order were not significant (*P* > 0.05)

^b^Comparisons between treatments were performed using the non-parametric Friedman Test for related samples

**Fig. 3:**
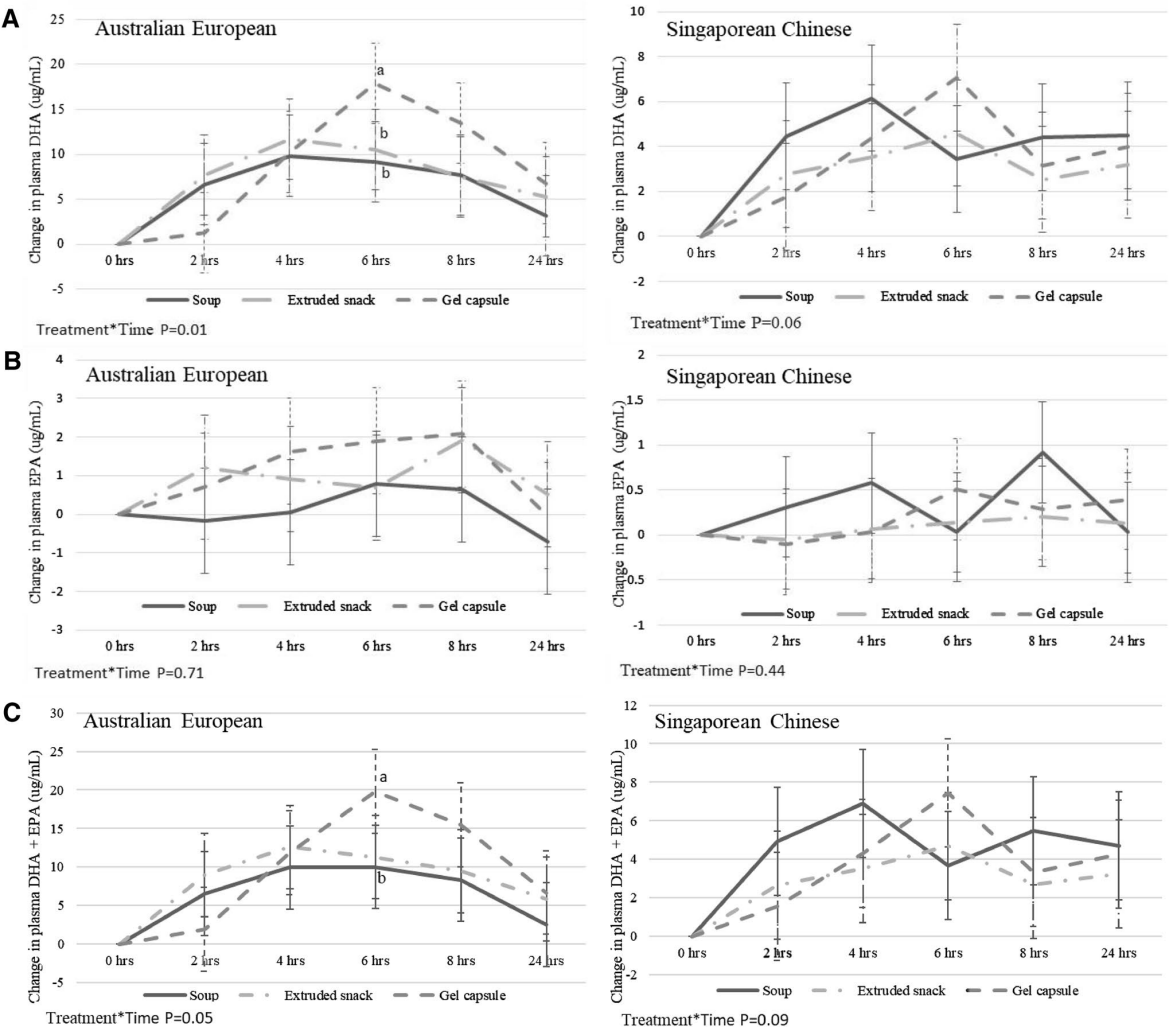
Adjusted mean (95% CI) postprandial changes in plasma DHA (**A**), EPA (**B**), DHA + EPA (**C**). DHA, docosahexaenoic acid; EPA, eicosapentaenoic acid; hrs, hours. Changes from Time_0_ were calculated by subtracting Time_0_ data from 2-, 4-, 6-, 8- and 24-h data. Comparisons between treatments were performed using linear mixed effects models. As significant treatment*time*ethnicity interactions were seen (DHA, *P* = 0.001 (**A**); EPA, *P* = 0.03 (**B**); DHA + EPA, *P* = 0.003 (**C**)), the results are presented separately for Australian European and Singapore Chinese groups. Time_0_ for the respective variables and treatments were included as confounder. Treatment*time*allocation order interaction was not significant (*P* > 0.05). ^a, b^Different symbols indicate significant differences between treatments. Post-hoc analysis were performed if significant treatment*time interactions were observed using repeated measures general linear model with Bonferroni adjustments.

Descriptive statistics estimated from raw data for plasma fatty acid concentrations over 24 h for the total group and stratified for ethnic groups are summarised in **Supplemental **Table 2. Adjusted mean (95% CI) postprandial changes in plasma DHA, EPA, DHA + EPA over 24 h between study products differed significantly between ethnic groups (treatment*time*ethnicity interaction, P < 0.05) (**Fig. ****3**). In the Australian European group, gel capsules resulted in significantly higher plasma DHA and DHA + EPA concentrations at 6 h compared to soup and extruded rice snack (for DHA) and soup (for EPA + DHA) while no significant treatment*time effects were seen in the Singaporean Chinese group. A similar trend (P = 0.06) was seen though for plasma DHA between gel capsules and soup at 6 h in the Singaporean Chinese group. The differences at 6 h were a result of a slight delay in increases in plasma DHA concentrations from gel capsules, causing peak concentrations to be reached later (at 6 h) compared to soup and extruded rice snack that started to decrease after 4 h. Plasma EPA concentrations did not change significantly over time, as expected considering that the test products contained minimal amounts of EPA.

### Adverse event

No adverse events were reported over the duration of the trial that were related to the study products.

## Discussion

This randomized controlled acute crossover trial was the first to investigate the bioavailability of omega-3 LCPUFA from foods fortified with a vegetable-based omega-3 LCPUFA delivery system. It was also the first study to investigate inter-ethnic differences in bioavailability of omega-3 LCPUFA. Bioavailability was assessed by calculating the incremental area under the postprandial curve over a 24-h period (iAUC_0-24 h_) after consumption of 2 test products (extruded rice snack and soup) containing vegetable (cauliflower)-encapsulated algal oil compared to a control (algal oil gel capsules) in Australian European and Singaporean Chinese populations. We showed that bioavailability as assessed by iAUC_0-24 h_ for plasma DHA, EPA and DHA + EPA were similar for extruded rice snack and soup compared to gel capsules. Furthermore, the structure of the test products i.e. solid (extruded rice snack) *vs.* semi-solid (soup) did not affect omega-3 LC PUFA bioavailability. Finally, bioavailability outcomes were similar between Australian European and Singaporean Chinese populations.

Evidence for the effect of food fortification delivery systems on omega-3 LCPUFA bioavailability is limited and there is no evidence for the effect of a vegetable-based delivery system. Microencapsulation of fish oil, a commonly used delivery system to incorporate omega-3 LCPUFA into foods, was shown to result in equivalent uptake of ~ 1 g EPA + DHA (delivered as powder taken with flavoured milk) into plasma compared to fish oil gel capsules post-prandially (over 48 h) and after 4 weeks of consumption [19]. Likewise, other studies delivering microencapsulated omega-3 LCPUFA in milkshakes [22], bread, biscuits and soup [24], or ready-to-eat meals [23] for 4 weeks showed similar bioavailability compared to fish oil capsules [22, 24] or liquid fish oil enriched ready-to-eat meals [23]. Some evidence suggests that combining the consumption of omega-3 LCPUFA with antioxidant-rich supplements may result in greater increases in circulatory levels. In this regard, Dams et al. [28] showed that plasma DHA levels were significantly higher when omega-3 LCPUFA (500 mg/day) supplements were taken together with a fruit, vegetable, and berry juice powder blend concentrate capsules for 16 weeks. Furthermore, Pipingas et al*.* [29] showed that fish oil consumed for 16 weeks combined with a multivitamin increased O3I compared to the same dose of fish oil. The authors speculated that the antioxidants in the supplements may have protected omega-3 LCPUFA from oxidation [29]. On the other hand, the presence of fibre in the matrix may impact bioavailability through trapping and excretion of fatty acids within the fibre matrix. Although evidence for the impact of fibre on omega-3 LCPUFA is lacking, inferences from other food matrixes high in fibre such as nuts that showed increased faecal fat loss, suggest that this is possible [43]. Thus, bioavailability of omega-3 LCPUFA from the vegetable-based delivery system, that is high in fibre and antioxidants, could have gone either way, increase or decrease, depending on the relative effect of antioxidant protection *vs.* potential trapping in fibre, but the results showed similar bioavailability compared to gel capsules.

The structure of the test products, in the current study, i.e. solid (extruded rice snack) *vs.* semi-solid (soup) did not have a significant effect on the bioavailability of EPA and DHA. Studies investigating the impact of food structure on omega-3 LCPUFA bioavailability have been inconsistent and results seem to differ between acute (single meal) studies *vs*. long-term studies where a steady state in omega-3 LCPUFA might have been achieved [44]. Sausages enriched with EPA and DHA (250 mg/day) consumed daily for 8 weeks resulted in O3I levels greater than previously reported for other sources [45]. Studies comparing fish *vs*. fish oil capsules showed similar changes in plasma/plasma phospholipid/erythrocyte omega-3 LC PUFA levels after chronic (2–16 weeks) consumption [25–27]. In contrast, Nasef, et al*.* showed that fish matrix may affect bioavailability of EPA and DHA post-prandially [44]. They compared the bioavailability of EPA and DHA from intact salmon (intact structure) with minced salmon (some structure) and defatted salmon flour combined with salmon oil (no structure) in an acute study over 6 h involving healthy female subjects. They observed significantly higher postprandial plasma EPA and DHA concentrations after participants consumed the intact salmon meal compared to the defatted salmon meal [44]. Results from an in vitro digestion study, that was performed in parallel, suggested that these differences may be ascribed to slower release of EPA and DHA from intact salmon as it requires hydrolysis of salmon protein to release salmon oil, whilst lack of structure may cause faster lipid transit out of the stomach resulting in less time for absorption of EPA and DHA in the intestine [44]. Limitations of this study were the short timeframe of 6 h, as plasma EPA and DHA were still increasing at the 6-h sampling point; and the EPA and DHA content in the defatted meal was lower than that of the intact salmon meal. In another 6-h acute study in healthy men, bioavailability of omega-3 LCPUFA, as measured by fatty acid composition of chylomicrons, was higher after consumption of omega-3 LCPUFA fortified drinking yoghurt compared to fortified fitness bar or fish oil capsules [46]. The authors attributed the greater bioavailability from yoghurt to pre-emulsification [46]. Raatz et al. showed enhanced bioavailability of EPA from emulsified fish oil preparations compared to capsular triacylglycerol over 48-h after a single dose [31]. The study by Schram et al*.*, however, had some critical limitations; chylomicron EPA and DHA levels were still on the rise after 6 h and it cannot be ruled out that bioavailability may have amounted to similar levels than other food matrices if the study was extended over a longer period. In addition, the fitness bar resulted in significantly higher concentrations of plasma diene conjugation (marker of oxidation) which may have occurred during the production process [46] and thus, may have negatively affected chylomicron EPA and DHA levels.

Several other factors may affect bioavailability of omega-3 LCPUFA. Predictors of increased blood omega-3 LCPUFA levels included increased dose, lower baseline O3I, chemical form of supplements (e.g. triacylglycerol *vs.* ethyl esters; phospholipids *vs*. triacylglycerol) [32–34], lower body weight, female sex [32], and a background diet lower in omega-6 PUFA [30]. To our knowledge, no other study has directly compared inter-ethnic biomarker responses to omega-3 LCPUFA intake. Patel et al*.* [35] concluded from a systematic literature review that ethnicity and culture may impact the protective effects of omega-3 LCPUFA against CVD which appeared to be moderated by the efficiency with which omega-3 LCPUFA are taken up from dietary sources. Our trial provides some evidence that bioavailability was not modulated by ethnicity (Australian European vs. Singaporean Chinese), but conclusions are limited by the acute nature of the current trial and small sample size. A better understanding of factors, including ethnicity and food/ingredient matrix, affecting responses to increased omega-3 LCPUFA intakes will assist in the development of personalised approaches to assist consumers in achieving omega-3 status that is optimal for health.

The increase in plasma DHA response curve was slightly delayed after consumption of gel capsules compared to the extruded rice snack and soup. This effect may have been due to the small lipidic bolus in gel capsules resulting in ineffective activation of lipid digestion and absorption processes, whilst the food form may be in a diluted emulsion as micro sized droplets, resulting in a higher surface area which may aid digestion and absorption processes [47, 48]. Furthermore, the fat content of a meal has been shown to affect EPA and DHA absorption with greater absorption from fish oil when consumed with a high-fat meal compared to a low-fat meal likely due to the stimulating effect of fat on the release of pancreatic lipase [49]. The extruded rice snack and soup test products had a higher fat content than gel capsules which may have contributed to delayed lipid digestion of gel capsules. However, as overall bioavailability, assessed by iAUC_0-24 h_, did not differ between test products, this effect is probably of little clinical importance.

Strengths of the study include the randomized controlled cross-over study design minimizing the effects of inter-individual variability in omega-3 LCPUFA bioavailability between study products. We provided a realistic and achievable omega-3 LCPUFA dose that fell within daily recommended ranges [14, 16]. We controlled for potential factors that may affect bioavailability (as mentioned above); we included participants with low habitual intake of omega-3 LCPUFA and strictly controlled the omega-3 LCPUFA and lipid content of meals during the 24-h postprandial period. Thus, changes in plasma omega-3 LCPUFA concentrations could only have been attributed to the single administration of the test products rather than other dietary fat or omega-3 LCPUFA sources. Other factors controlled for in inclusion criteria were age, BMI and gender (restricted to males).

The study also had limitations including using a single-dose postprandial approach which gives an indication of the omega-3 LCPUFA that reaches the circulation in the short-term, but does not determine the extent to which omega-3 LCPUFA are taken up into target tissues from where it normally exerts its physiological effects [21]. Despite the rigorously controlled study design, large inter-individual responses to study products were seen, consistent with findings from Köhler et al*.* [50]. While the rigorously controlled study design was required to minimize the effects of factors on bioavailability, it restricts generalizability of the results. To confirm bioequivalence between the vegetable-based omega-3 LCPUFA fortified extruded rice snack and soup compared to gel capsules, a longer-term trial of at least 16 weeks, in a broader sample, that assesses erythrocyte DHA and EPA levels which is a suitable long-term biomarker for tissue uptake and allows calculation of the O3I, is required [10].

In conclusion, the vegetable-based omega-3 LCPUFA delivery system did not affect bioavailability of omega-3 LCPUFA in healthy young Australian and Singaporean men as assessed after a single meal over 24 h. Furthermore, bioavailability did not differ between Australian European and Singaporean Chinese populations. This novel delivery system may be an effective way to fortify foods/beverages with omega-3 LCPUFA providing consumers with alternative sources to increase their omega-3 LCPUFA intake. In addition, if a vegetarian omega-3 LCPUFA oil is used within the delivery system, such as algal oil in the current study, the products will be suitable for vegans and vegetarians, a population particularly at risk of omega-3 LCPUFA deficiency. The delivery system may also help to simultaneously augment the vegetable intake of consumers contributing to better health outcomes.

## Supplementary Information

Below is the link to the electronic supplementary material.

Supplementary file1 (PDF 282 KB)

## Data Availability

Data described in the manuscript, code book, and analytic code will be made available upon request pending application and approval.

## Abbreviations

iAUC: Incremental area under the curve
BIA: Bioelectrical impedance analysis
BMI: Body mass index
CHD: Coronary heart disease
CVD: Cardiovascular disease
DHA: Docosahexaenoic acid
EPA: Eicosapentaenoic acid
FAME: Fatty acid methyl esterification
GC: Gas chromatography
HACCP: Hazard Analysis Critical Control Point
ICH-GCP: International Council for Harmonisation Good Clinical Practice
LCPUFA: Long-chain polyunsaturated fatty acids
NCD: Non-communicable disease
O3I: Omega-3 index
