# Supplementary material for: Bioequivalence of long-chain omega-3 polyunsaturated fatty acids from foods enriched with a novel vegetable-based omega-3 delivery system compared to gel capsules: a randomized controlled cross-over acute trial
Source: Eur J Nutr. 2022 Jan 18;61(4):2129–41. doi: 10.1007/s00394-021-02795-7 (PMC9106597; doi:10.1007/s00394-021-02795-7)
Supplement: Supplementary file 1 — Supplementary file1 (PDF 282 KB) [file 394_2021_2795_MOESM1_ESM.pdf]

Supplemental Information

Bioequivalence of long-chain omega-3 polyunsaturated fatty acids from foods enriched with a novel vegetable-based omega-3 delivery system compared to gel capsules – a randomized controlled cross-over acute trial

Welma Stonehouse[1], Bradley Klingner[1], Rachel Tso[2], Pey Sze Teo[2], Netsanet Shiferaw Terefe[3], Ciarán G. Forde[2,4†]

[1]Commonwealth Scientific Industrial Research Organisation (CSIRO), Health and Biosecurity, Adelaide, South Australia, Australia

[2]Clinical Nutrition Research Centre, A*STAR Singapore Institute of Food and Biotechnology Innovation, Singapore

[3]Commonwealth Scientific Industrial Research Organisation (CSIRO), Agriculture and Food, Werribee, Victoria, Australia

[4]Department of Physiology, Yong Loo Lin School of Medicine, National University of Singapore, Singapore

†Current address: Wageningen University, Sensory Science and Eating Behaviour, Division of Human Nutrition and Health, Wageningen, The Netherlands.

Supplemental Table 1. Descriptive statistics for plasma DHA, EPA and DHA + EPA $iAUC_{0\text{-}24h}$, Cmax and Tmax for the total group and stratified for ethnic groups

Variable		Soup			Extruded rice snack			Gel capsule		
**Total group (n=27)**										
DHA $iAUC_{0\text{-}24h}$ (µg/mL*hour)	Mean (SD)	8069	5500		7367	5599		9864	8603	
	Median (25, 75 Percentile)	8568	3752	10999	6949	3059	9619	8111	5123	12250
DHA Cmax (µg/mL)	Mean (SD)	52.5	25.6		51.1	25.6		55.6	28.1	
	Median (25, 75 Percentile)	45.7	35.7	65.4	40.4	30.3	73.6	42.9	36.9	78.9
DHA Tmax (hour)	Mean (SD)	7.19	7.47		5.56	5.69		7.41	6.12	
	Median (25, 75 Percentile)	4.00	2.00	8.00	4.00	2.00	6.00	6.00	4.00	6.00
EPA $iAUC_{0\text{-}24h}$ (µg/mL*hour)	Mean (SD)	1192	1242		1241	1748		1331	1587	
	Median (25, 75 Percentile)	982	19	1753	486	125	1446	976	143	1628
EPA Cmax (µg/mL)	Mean (SD)	18.2	13.0		18.7	15.4		18.7	15.9	
	Median (25, 75 Percentile)	14.6	6.90	28.2	9.07	5.78	32.2	8.16	6.04	28.5
EPA Tmax (hour)	Mean (SD)	6.15	6.02		8.96	8.51		8.67	7.81	
	Median (25, 75 Percentile)	6.00	2.00	8.00	6.00	2.00	8.00	6.00	4.00	8.00
DHA + EPA $iAUC_{0\text{-}24h}$ (µg/mL*hour)	Mean (SD)	8872	6626		8396	7022		11054	9886	
	Median (25, 75 Percentile)	7627	4169	14129	7737	2902	10894	9059	5243	14390
DHA + EPA Cmax (µg/mL)	Mean (SD)	70.2	36.8		69.4	39.6		73.9	42.7	
	Median (25, 75 Percentile)	59.2	41.7	107	47.8	34.1	94.3	50.9	42.0	107
DHA + EPA Tmax (hour)	Mean (SD)	6.81	6.62		6.89	7.53		7.26	6.28	
	Median (25, 75 Percentile)	4.00	4.00	8.00	4.00	2.00	6.00	6.00	4.00	6.00
**Australian Europeans (n=12)**										
DHA $iAUC_{0\text{-}24h}$ (µg/mL*hour)	Mean (SD)	10158	6669		10387	6540		14110	10846	
	Median (25, 75 Percentile)	10144	3212	16756	9310	6382	12235	10590	7115	17286
DHA Cmax (µg/mL)	Mean (SD)	74.0	23.3		74.1	21.0		81.0	22.7	
	Median (25, 75 Percentile)	74.6	54.6	87.8	77.5	54.8	91.2	81.7	61.9	94.1
DHA Tmax (hour)	Mean (SD)	4.50	2.43		3.83	1.80		7.17	5.42	
	Median (25, 75 Percentile)	4.00	2.00	7.00	4.00	2.00	4.00	6.00	5.00	6.00
EPA $iAUC_{0\text{-}24h}$ (µg/mL*hour)	Mean (SD)	1642	1638		2112	2231		1943	2179	
	Median (25, 75 Percentile)	1204	64	2949	1050	346	4033	1127	178	3510
EPA Cmax (µg/mL)	Mean (SD)	29.9	9.19		34.3	8.61		34.2	10.5	
	Median (25, 75 Percentile)	28.4	23.0	36.8	33.6	28.0	40.8	31.3	25.4	41.2
EPA Tmax (hour)	Mean (SD)	5.00	3.46		9.67	8.94		5.83	2.33	
	Median (25, 75 Percentile)	6.00	1.00	8.00	7.00	3.00	16.00	6.00	4.00	8.00
DHA + EPA $iAUC_{0\text{-}24h}$ (µg/mL*hour)	Mean (SD)	11204	8258		12123	8385		15783	12615	
	Median (25, 75 Percentile)	13293	3287	19267	10882	7192	13288	12126	6825	18214
DHA + EPA Cmax (µg/mL)	Mean (SD)	103	29.0		108	26.5		115	30.4	
	Median (25, 75 Percentile)	107	79.2	119	106	87.7	132	109	94.0	131
DHA + EPA Tmax (hour)	Mean (SD)	5.00	2.49		3.83	1.80		7.33	5.42	
	Median (25, 75 Percentile)	4.00	3.00	8.00	4.00	2.00	4.00	6.00	5.00	7.00
**Singaporean Chinese (n=15)**										
DHA $iAUC_{0\text{-}24h}$ (µg/mL*hour)	Mean (SD)	6397	3809		4951	3246		6468	4149	
	Median (25, 75 Percentile)	5169	4101	9918	4897	2081	7924	7740	3084	8780
DHA Cmax (µg/mL)	Mean (SD)	35.4	9.34		32.6	7.29		35.2	7.55	
	Median (25, 75 Percentile)	36.8	29.0	38.6	31.2	28.3	38.8	38.7	27.1	41.7
DHA Tmax (hour)	Mean (SD)	9.33	9.37		6.93	7.28		7.60	6.81	
	Median (25, 75 Percentile)	4.00	4.00	24.00	6.00	2.00	6.00	6.00	4.00	6.00

EPA $iAUC_{0-24h}$ (μg/mL*hour)	Mean (SD)	832	668		545	773		841	610	
	Median (25, 75 Percentile)	982	19	1339	278	37	632	976	75	1301
EPA Cmax (μg/mL)	Mean (SD)	8.73	5.69		6.26	2.68		6.26	2.97	
	Median (25, 75 Percentile)	7.28	5.48	10.9	6.67	4.17	7.83	6.16	4.06	7.44
EPA Tmax (hour)	Mean (SD)	7.07	7.48		8.40	8.42		10.93	9.82	
	Median (25, 75 Percentile)	6.00	2.00	8.00	6.00	2.00	8.00	6.00	2.00	24.00
DHA + EPA $iAUC_{0-24h}$ (μg/mL*hour)	Mean (SD)	7006	4422		5415	3859		7271	4689	
	Median (25, 75 Percentile)	5667	4400	10628	4864	2063	8310	9027	3002	10021
DHA + EPA Cmax (μg/mL)	Mean (SD)	44.0	14.1		38.8	9.51		41.3	9.63	
	Median (25, 75 Percentile)	43.0	32.9	53.0	35.0	33.0	47.0	44.1	32.5	47.9
DHA + EPA Tmax (hour)	Mean (SD)	8.27	8.45		9.33	9.40		7.20	7.08	
	Median (25, 75 Percentile)	4.00	4.00	8.00	6.00	2.00	24.00	6.00	4.00	6.00

DHA, docosahexaenoic acid; EPA, eicosapentaenoic acid; $iAUC_{0-24h}$; incremental area under the curve over 24 hours; Cmax, maximal concentration; Tmax, time to maximal concentration
Descriptive statistics were calculated from raw data.

Supplemental Table 2. Descriptive statistics for plasma fatty acid concentrations over 24 hours for the total group and stratified for ethnic groups

Variable	Time (hrs)	Total Group						Australian European						Singaporean Chinese					
		Soup		Extruded rice snack		Gel capsule		Soup		Extruded rice snack		Gel capsule		Soup		Extruded rice snack		Gel capsule	
		Mean	SD	Mean	SD	Mean	SD	Mean	SD	Mean	SD	Mean	SD	Mean	SD	Mean	SD	Mean	SD
DHA (ug/mL)	0	41.1	21.3	40.6	21.6	41.3	22.5	59.5	17.8	59.0	18.9	61.4	18.0	26.4	8.22	25.8	7.66	25.3	8.01
	2	46.5	21.5	45.6	23.7	42.9	21.5	66.2	15.8	66.8	19.5	62.6	16.3	30.6	7.70	28.6	7.11	27.2	7.44
	4	48.8	23.7	47.7	26.1	48.4	24.5	69.4	20.0	70.7	22.4	71.6	17.4	32.4	8.60	29.4	7.21	29.9	6.92
	6	47.0	25.4	47.9	24.8	53.3	28.4	68.7	22.8	69.6	21.9	79.2	22.8	29.7	8.07	30.4	5.74	32.5	6.81
	8	46.9	24.0	45.3	23.1	49.1	27.4	67.2	20.8	66.6	17.9	74.8	20.7	30.6	9.43	28.4	6.57	28.6	6.62
	24	44.9	21.0	44.7	22.1	46.6	24.6	62.7	18.5	64.3	17.9	68.1	21.0	30.7	7.62	29.0	7.65	29.5	8.38
EPA (ug/mL)	0	16.3	12.4	16.9	14.1	16.7	14.7	27.6	9.01	31.2	8.00	31.3	9.23	7.30	4.89	5.54	2.63	5.06	2.57
	2	16.3	12.0	17.5	14.8	17.0	15.0	27.4	8.39	32.4	8.34	32.0	9.35	7.43	4.45	5.55	2.54	5.08	2.33
	4	16.6	11.9	17.4	14.7	17.5	15.5	27.7	8.46	32.1	9.05	32.9	9.99	7.70	4.33	5.67	2.60	5.22	2.38
	6	16.6	12.7	17.4	14.5	17.9	15.5	28.4	9.19	31.9	8.76	33.2	10.0	7.16	4.00	5.74	2.39	5.69	2.40
	8	17.0	12.5	17.9	14.9	17.9	15.7	28.3	8.98	33.1	8.19	33.3	10.2	8.04	5.73	5.80	2.67	5.47	2.35
	24	15.9	11.8	17.3	14.3	17.0	14.4	26.9	8.88	31.7	8.14	31.2	9.03	7.15	3.49	5.73	2.72	5.58	3.02
DHA + EPA (ug/mL)	0	57.4	32.3	57.5	34.2	58.1	36.0	87.1	23.2	90.2	23.4	92.7	24.3	33.7	12.3	31.4	9.86	30.4	9.96
	2	62.8	32.0	63.1	37.2	60.0	35.4	93.7	19.8	99.2	24.6	94.6	22.3	38.1	11.0	34.2	9.07	32.3	9.15
	4	65.4	34.3	65.2	39.7	65.9	39.1	97.1	25.3	103	28.7	104.5	24.4	40.1	11.6	35.0	9.33	35.1	8.48
	6	63.6	36.8	65.2	38.3	71.2	42.9	97.1	29.1	101	28.5	112.4	30.3	36.8	11.0	36.2	7.46	38.2	8.23
	8	63.9	35.1	63.3	36.8	67.0	42.2	95.5	26.2	99.7	22.4	108.1	28.4	38.6	14.5	34.2	8.85	34.1	8.42
	24	60.9	31.7	62.0	35.2	63.6	37.9	89.6	24.6	96.0	23.0	99.3	27.2	37.8	10.5	34.8	10.0	35.0	10.9

DHA, docosahexaenoic acid; EPA, eicosapentaenoic acid.
Descriptive statistics were calculated from raw data.

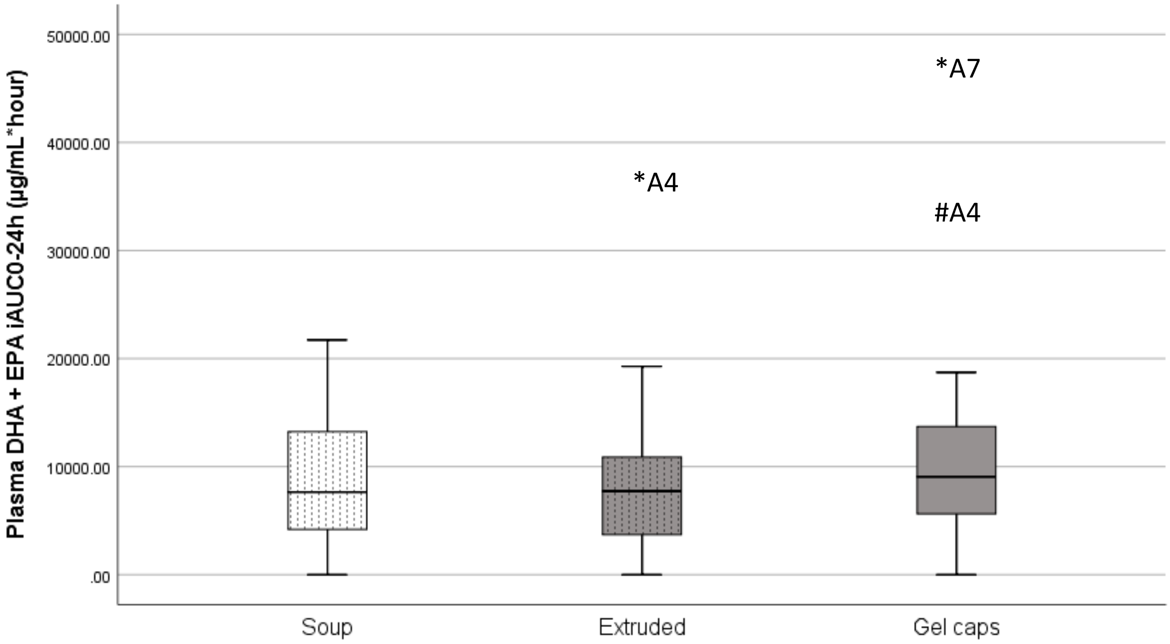

Supplemental Figure 1. Box plots of DHA + EPA $iAUC_{0\text{-}24h}$ by treatment.
DHA, docosahexaenoic acid; EPA, eicosapentaenoic acid; $iAUC_{0\text{-}24h}$; incremental area under the curve over 24 hours.
#Outlier (1.5-3 box lengths); *Extreme outlier (>3 box lengths); A4, A7, Australian European participants number 4 and 7.

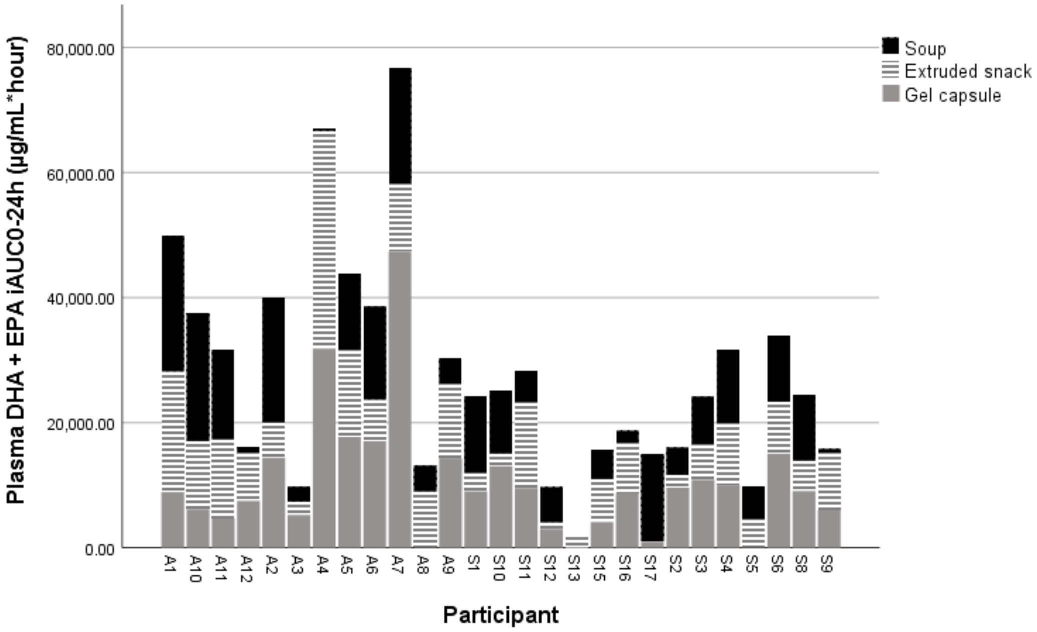

Supplemental Figure 2. Individual participant's incremental area under the curve ($iAUC_{0\text{-}24hr}$) for plasma DHA + EPA (C) by treatment.
DHA, docosahexaenoic acid; EPA, eicosapentaenoic acid; $iAUC_{0\text{-}24h}$; incremental area under the curve over 24 hours.
Participants numbered A1-12 = Australian European; S1=17 = Singaporean Chinese
